# iMODS: internal coordinates normal mode analysis server

**DOI:** 10.1093/nar/gku339

**Published:** 2014-04-25

**Authors:** José Ramón López-Blanco, José I. Aliaga, Enrique S. Quintana-Ortí, Pablo Chacón

**Affiliations:** 1 Department of Biological Chemical Physics, Rocasolano Physical Chemistry Institute C.S.I.C., Serrano 119, 28006 Madrid, Spain; 2 Department of Computer Science and Engineering, University Jaume I, 12071 Castellón, Spain

## Abstract

Normal mode analysis (NMA) in internal (dihedral) coordinates naturally reproduces the collective functional motions of biological macromolecules. iMODS facilitates the exploration of such modes and generates feasible transition pathways between two homologous structures, even with large macromolecules. The distinctive internal coordinate formulation improves the efficiency of NMA and extends its applicability while implicitly maintaining stereochemistry. Vibrational analysis, motion animations and morphing trajectories can be easily carried out at different resolution scales almost interactively. The server is versatile; non-specialists can rapidly characterize potential conformational changes, whereas advanced users can customize the model resolution with multiple coarse-grained atomic representations and elastic network potentials. iMODS supports advanced visualization capabilities for illustrating collective motions, including an improved affine-model-based arrow representation of domain dynamics. The generated all-heavy-atoms conformations can be used to introduce flexibility for more advanced modeling or sampling strategies. The server is free and open to all users with no login requirement at http://imods.chaconlab.org.

## INTRODUCTION

The main functions of living cells (replication, transcription, translation, folding and protein turnover) are usually governed by large macromolecular complexes (polymerases, ribosomes, chaperonins and proteasomes). The mechanisms of action of these complexes are usually dynamical and entail large and collective conformational changes. Thus, efficient tools for the easy and rapid modeling of such motions are in high demand. Normal mode analysis (NMA) is a popular approach for describing the collective functional motions of such macromolecules (1–3). Each normal mode comprises both a deformation vector and a frequency. The former codifies an atomic displacement direction and the latter is related to the relative motion amplitude. NMA in Cartesian coordinates has been used for many years in online modeling of protein flexibility. For example, the servers AD-ENM, DFprot, ElNémo, HingeProt, MolMovDB, NOMAD-Ref, TMM@, oANM and WEBnm@ employ this methodology (for details, see review (4)). The PATH-ENM (5), iENM (6), NMSim (7), KOSMOS (8) and FlexServ servers (9) also rely on NMA to generate transition pathways between two different conformations. NMA in dihedral space is a more natural and effective approach than the Cartesian formulation for modeling macromolecular conformational changes. For example, in a recent work, Levitt *et al.*, using a set of 13 proteins in two different conformational states, showed that modes in internal coordinates (torsion angles) reproduce protein conformational changes more accurately than Cartesian approximations (10). Furthermore, we have shown how approaches based on Cartesian NMA can accumulate important geometrical distortions when used for the flexible fitting of atomic structures into electron microscopy maps (11). The animation of Cartesian modes can produce non-physical distortions in bond length and angles because atoms move along straight lines. In contrast, working in internal coordinates, the covalent bonding geometry is implicitly preserved, avoiding such potential geometrical distortions. Moreover, the internal coordinate method requires at least one-third fewer degrees of freedom and hence leads to a substantial increase in the calculation speed and the ability to handle larger systems. Here, we present a new server based on our NMA in the internal coordinates package iMod (12). Several optimizations have been applied to iMod for supporting online service. First, the method has been improved, including the implementation of a faster eigenproblem solver (13). This improvement permitted the computation of the lowest-frequency modes almost interactively for standard-size systems while extending the application range to large macromolecules. Second, an affine-model-based approach (14) has been implemented to facilitate normal mode visualization. This approach effectively identifies those atoms moving together as rigid bodies and depicts their motion with simple curved arrows. Finally, the simulation of conformational transition trajectories has been extended to address homologous structures.

The iMod server (iMODS) provides a user-friendly interface for this enhanced NMA methodology in internal coordinates. The web interface is very intuitive and responsive to all major browsers and even to modern mobile appliances. Users can perform NMA or simulate feasible trajectories between two conformations and interactively explore in 3D the resulting structures, animations and trajectories, even for large macromolecules. The generated conformations and pathways can be downloaded and used as inputs in more sophisticated sampling or modeling techniques.

## MATERIALS AND METHODS

### Normal mode analysis in internal coordinates

The methodology underlying iMODS has been detailed elsewhere (12). Briefly, the molecular system is modeled as a set of atoms connected by harmonic springs. The potential energy is given by

(1) \begin{equation*} V = \sum\limits_{i {<} j} {F_{ij} (r_{ij}^t - r_{ij}^0 )^2 } , \end{equation*}

where *F_ij_* is the spring stiffness matrix; *r_ij_* is the distance between atoms *i* and *j*; and *t* and *0* are the current and equilibrium conformations, respectively. The modes are computed from the Lagrangian equations of motion by solving the corresponding generalized eigenvalue problem (15):

(2) \begin{equation*} {\bf HX = \Lambda TX}, \end{equation*}

where **H** is the Hessian or second derivative of the potential, **T** represents the kinetic energy, matrix **X** = (**x**_1_, **x**_2_, …, **x***_n_*) contains the eigenvectors and **Λ** = diag(*λ*_1_, *λ*_2_, …, *λ_n_*) is a diagonal matrix with the eigenvalues of the problem. The general solutions in internal coordinates, *q*, have the following form:

(3) \begin{equation*} q_k^t = q_k^0 + \sum\limits_{k = 1}^n {a_k {\bf x}_k \cos (2\pi v_k t + \delta _k )} , \end{equation*}

where *a_k_* and *δ_k_* depend on the initial conditions, **x***_k_* is the *k*th eigenvector and *v_k_* its associated frequency. These eigenvectors, or normal modes, are a set of orthogonal displacements. The high-frequency modes represent localized displacements, whereas low-frequency modes correspond to collective conformational changes. The solution to equation 2 is the computational bottleneck in NMA. To address this problem, the LAPACK-based eigensolver used by Lopez-Blanco *et al*. (12) has been replaced by a new parallel implementation of the iterative Krylov-subspace method (13). This method is especially appropriate when only the lowest-frequency modes are required. Indeed, this alternative allows for rapid NMA calculations, enhancing server interactivity. For example, the computation of the first 50 modes of a 1000 amino acid protein takes only three seconds on our server.

### Affine model-based arrow representation

**Figure 1.**
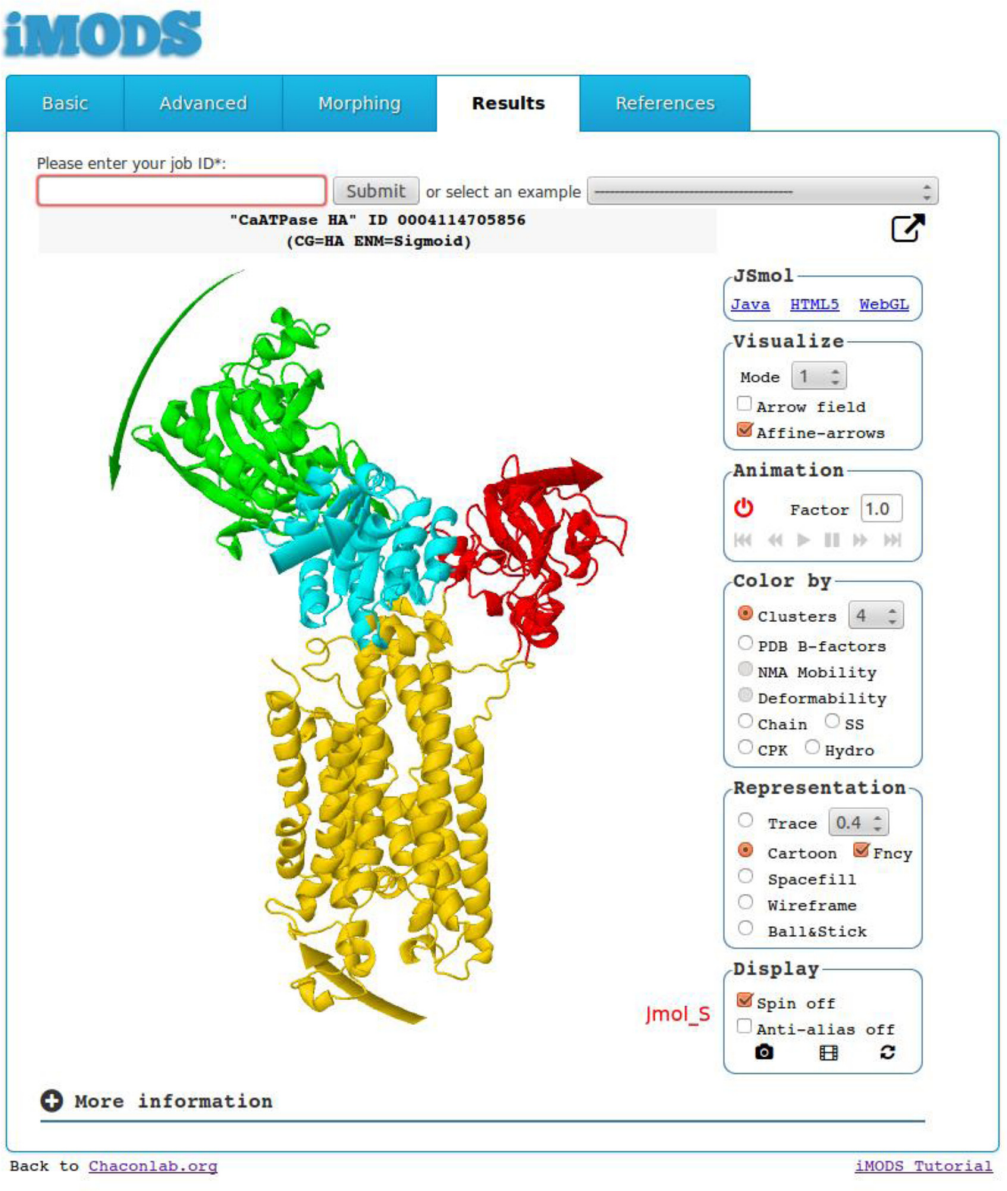
Screenshot of iMODS results obtained from the Ca^2+^-ATPase structure. The center frame displays the functional domain motions encoded by the lowest-frequency mode. The actuator (A), phosphorylation (P), nucleotidic (N) and transmembrane (M) functional domains appear in red, cyan, green and yellow, respectively. The arrows obtained with the affine-model-based approach are colored accordingly.

The mode visualization has been enhanced by clustering those atoms moving together as rigid bodies according to a given affine transformation (14). In this way, the domain motions encoded by a single normal mode can be summarized with a handful of curved arrows. The procedure comprises both clustering and arrow generation. First, the atomic modal displacements are clustered in different affine models using a hierarchical agglomerative algorithm. The clustering also delimits potential dynamical domains. Next, the affine model of every cluster is used to compute all possible paths near the macromolecular surface. Finally, the longest path is depicted as a wide curved arrow to represent the trajectory followed by each dynamical domain. The arrows and dynamical domains are colored accordingly to improve the visualization of the motions encoded in the mode, as illustrated in Figure 1. We optimized the original methodology to support online service, but this process still requires more time than the NMA calculation.

## DESCRIPTION OF THE WEB SERVER

Upon atomic structure submission or PDB ID (Protein Data Bank IDentification entry) fetching, iMODS (imods.chaconlab.org) can perform all of the following:

### Standard NMA in internal coordinates

From a single structure, the server calculates the lowest-frequency normal modes in internal coordinates. After the submit button is clicked, a results page appears almost immediately, where the user can interactively explore potential collective motions. The server provides several effective motion representations, including a vector field, affine-model arrows (14) (Figure 1) and modal animations. The detailed analysis includes profiles of mobility (NMA B-factors), deformability (16), eigenvalues, covariance map (17) and linking matrix. All these results are stored and can be retrieved at any time or downloaded as a compressed file.

### Feasible pathways between two conformations

To simulate feasible transitions, the initial structure is iteratively deformed along the lowest modes while the root mean square deviation (RMSD) to a target structure is minimized. Two initial superimposition methods can be selected: either global (18) or local (19). Whereas the former considers all atoms for the RMSD, the latter favors the overlap between most similar regions. Upon submission, the RMSD between the target and final structures can be interactively visualized. In a matter of minutes for relatively large molecules (∼1000 residues), the complete trajectory is ready for 3D interaction and downloading. For non-identical sequences, only homologous residues are matched in RMSD minimization.

### Versatility

iMODS is highly customizable; it admits several coarse-grained (CG) levels. In addition to the standard all-heavy atoms representation, protein structures can be coarse grained as one Cα or five pseudo-atoms per amino acid (12). The user can fix parts of the input molecule, including either secondary structure elements or dihedral angles at random. Freezing randomly dihedral angles in the NMA calculations is a simple and efficient CG strategy to speed up calculations without incurring a significant loss of accuracy (12). The system can also handle nucleic acids and small rigid ligands. Several elastic network potentials have been implemented, including the default inverse exponential (16), classical distance cut-off (20) and two MD-derived ENM potentials (21,22). Another advantage of the web application is its efficiency; NMA takes only a few seconds for a large protein. This also makes possible to perform the NMA of large systems without high-end resources.

## EXAMPLES OF USE

### Calcium ATPase

#### Normal mode analysis

The low-frequency modes of the sarco/endoplasmic reticulum Ca^2+^-ATPase (SERCA) can be readily obtained by submitting or fetching the open atomic structure (PDB ID: 1su4). In a few seconds, the highly dynamic nature of the cytoplasmic domains can be appreciated by exploring the animations of the first 10 modes. Other interesting detected motions include a transmembrane region twist that has been suggested as a possible exit path for Ca^2+^ (23) (at mode four) and the lumenal side opening (at modes 10–19). The new affine-model-based arrow representation emphasizes the direction of such functional motions (Figure 1). In addition, the affine-model clustering procedure constitutes an effective domain detection tool. When four clusters are requested for modes one and three, the dynamical domains detected agree well with the actuator (A), phosphorylation (P), nucleotidic (N) and transmembrane (M) functional domains (24). Note that the domain decomposition depends on the selected mode. However, as occurs in this case, it is likely to find valid domain decompositions by exploring the clusters of the first modes that typically encode the functional motions.

Several useful results are provided in addition to the direct visualization of mode motions. The NMA-derived B-factors (16) indicate the relative amplitude of the atomic displacements around the equilibrium conformation. To identify mobile or static regions, the 3D structure can be colored by these computed B-factors or they can be directly plotted and compared in 2D with the corresponding experimental values stored in the PDB file. In this example, in consonance with observed functional states (25) and other NMA studies (23,26), the NMA B-factors correctly identify N, A, the luminal part of M and the tip of P as highly dynamical domains. Although experimental and calculated B-factors were in good agreement, the mobility of the N domain was clearly underestimated by the crystallographic B-factors, exhibiting crystal-packing effects. Another interesting measure is the deformability (16). This metric computes the gradient (spatial first derivative) of the atomic displacements summed over all modes at every atomic position. High values are expected in flexible regions such as hinges or linkers between domains, whereas low values usually correspond to rigid parts. In SERCA case, the observed high deformability regions are compatible with previously reported flexible linkers between P and N and between M helices and A as well as between luminal transmembrane loops (23,26).

The analysis can be completed with more NMA-based measurements. Each mode fluctuates with amplitude inversely proportional to the eigenvalue. The magnitude of these fluctuations can be easily expressed as a variance to estimate the relative importance of the modes. In SERCA, ∼80% of the variance is justified by only the first seven modes. The correlated, uncorrelated or anti-correlated motions between dynamical regions can also be characterized by covariance analysis (17). Finally, the elastic network model utilized in NMA calculations can be directly visualized as a linking matrix.

#### Conformational transition

When calcium ions dissociate from the open form, the cytoplasmic domains and transmembrane helices reorganize into a closed form (25). Such large and collective motions are ideal candidates to be modeled by NMA. Upon fetching the initial (open, PDB ID: 1su4) and target structures (closed, PDB ID: 1iwo), the computation of the conformational transition pathway begins. In less than ∼30 minutes, the user can monitor how the Cα-RMSD smoothly converges to ∼2 Å from an initial value of 19 Å (local superimposition). When the calculation is completed, the feasible pathway linking both states can be interactively explored. The major rearrangements correspond to the cytoplasmic regions. The N domain motion experiences a simple ∼60° rotation toward the A domain around a virtual hinge located between the P and N domains. The A domain motion is more complex, as it combines translation with biaxial rotation. The N domain motion corresponds very well to the first mode, whereas the A domain motion can be described by a complex combination of the first modes. These collective motions were already present in the previous NMA from a single open structure. In addition to cytoplasmic motions, rearrangements in the M domain helices connecting with A domain are also observed. As local motions cannot be described by low-frequency modal space, the majority of the final RMSD deviation is typically concentrated in small loop regions. In any case, the feasibility of the generated pathways should be validated with experimental data.

In summary, the collective motions observed either in the normal modes or in the conformational transitions can provide useful insights regarding how calcium is translocated through the membrane. Similar conclusions can be derived using the different CG strategies implemented in iMODS.

### GroEL/ES complex

#### Normal mode analysis

**Figure 2.**
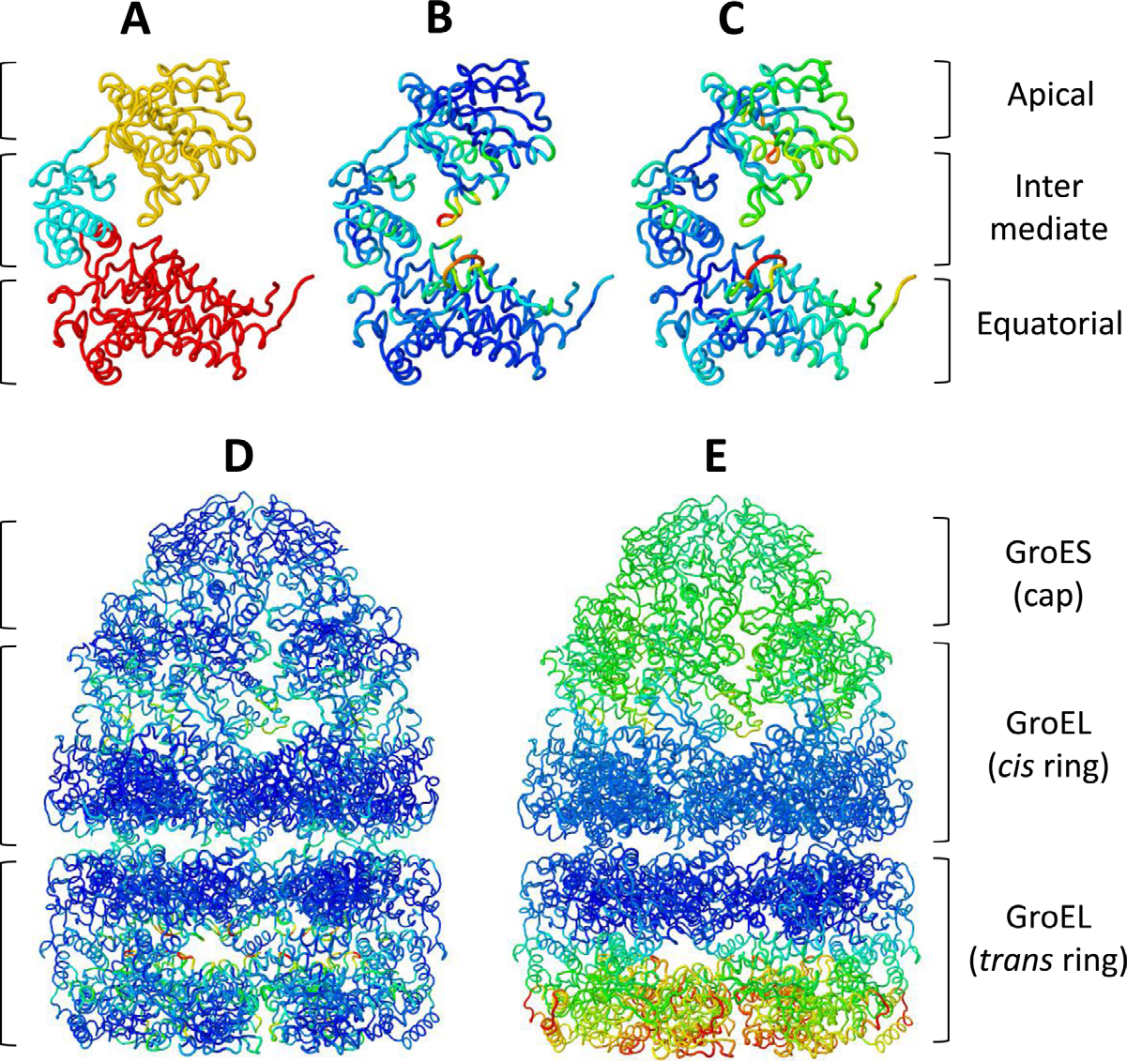
NMA of GroEL/ES complex. Affine model clusters computed from a single *trans* GroEL monomer (**A**). The characterized dynamical domains in yellow, cyan and red are in agreement with the apical, intermediate and equatorial functional domains, respectively. The GroEL monomer structure is colored as a function of deformability (**B**) and mobility (**C**). The GroEL/ES deformability (**D**) and mobility (**E**) were calculated from the NMA of the whole complex.

This bacterial chaperonin forms an oligomeric structure consisting of 14 GroEL subunits arranged in two inverted heptameric rings, one of them capped by a GroES heptamer. To illustrate iMODS performance, we carried out two NMAs, one with the GroEL subunit and a second with the whole complex. The three functional domains—equatorial (E), intermediate (I) and apical (A)—can be easily identified from the single GroEL monomer NMA with the help of affine model clustering (Figure 2A). There is strong evidence that these domains behave mainly as rigid bodies during the reaction cycle (27). The large collective displacements of the A and I domains taking place upon Adenosine triphosphate (ATP) and GroES binding to the *cis* ring are fairly well represented by the lowest modes of the monomer. The deformability calculations obtained from any of the monomers of both rings show that A and I are the most flexible domains together with the stem-loop (residues 38–50) of E (Figure 2B).

Using the complete GroEL/ES structure (8015 amino acids, PDB ID: 1sx4), the low-frequency modes are now dominated by the collective motions of the complete complex. These modes are particularly compatible with the cooperative motions involved in substrate internalization (modes 1 and 4) and GroES cap release (modes 1 and 9) (27). The deformability pattern of the complex (Figure 2D) agrees with that computed from the monomers (Figure 2B). However, the relative values observed in both rings are significantly higher in the uncapped ring (*trans*). This flexibility increment may be important for substrate internalization. Furthermore, only the NMA of the complete complex predicts high deformability in several inter- and intra-ring loops, which would be related to the cooperative mechanism observed upon ATP binding. The computed B-factors for the full structure predict that the E domains are mainly static, whereas A and I are highly mobile, particularly for the uncapped ring (Figure 2E). However, in the monomers, the E domain incorrectly appears as very mobile (Figure 2C). These results highlight the importance of considering adequate structures to extract meaningful information from NMA. In any case, all results are consistent with previous NMA (28), targeted molecular dynamics (29) and crystallographic anisotropic displacement studies (27).

**Figure 3.**
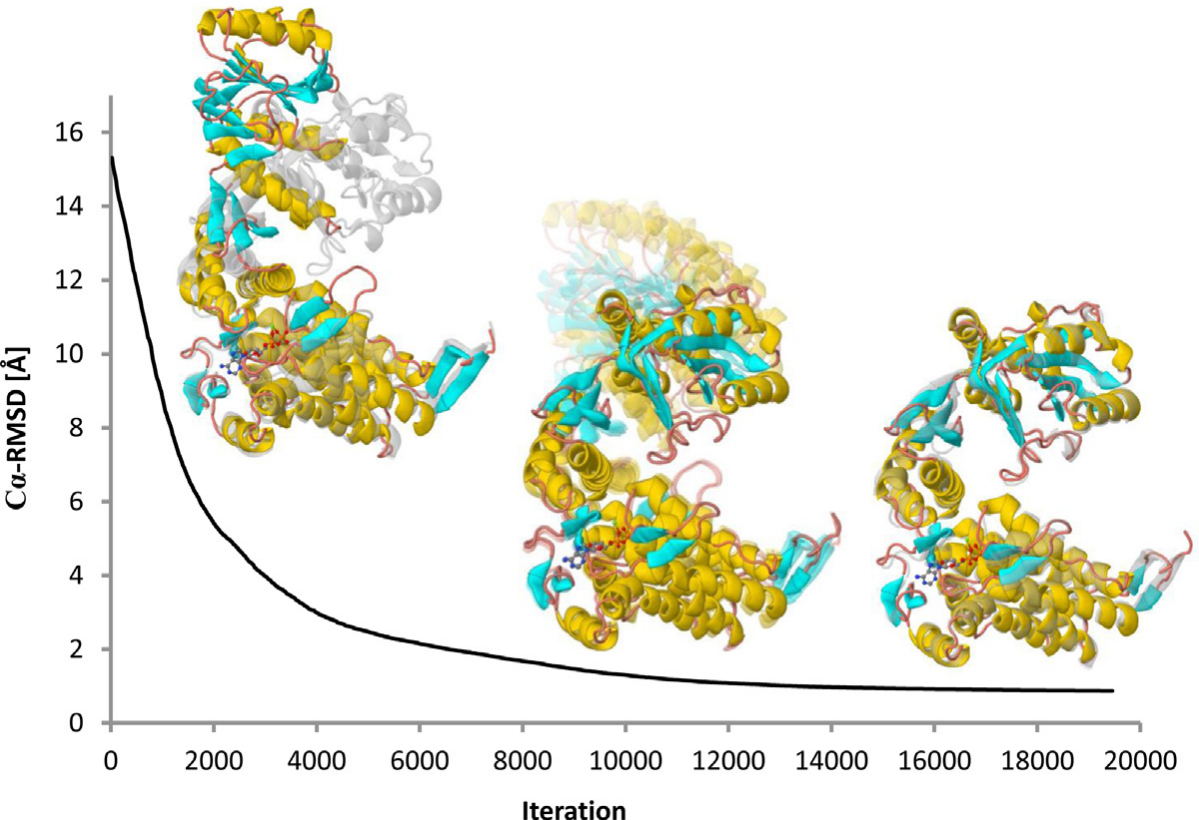
GroEL monomer *cis*/*trans* transition. The trajectory of the large conformational change between the open (*cis*) and closed (*trans*) structures. Initial (left) and final (right) conformations are illustrated superimposed with the target structure in grey. Notice how the Cα-RMSD between the trajectory models and the target structure smoothly converges to ∼1 Å. All-heavy-atoms were considered, including the ATP/Mg^2+^ ligand. All of the figures were generated from images provided by iMODS.

#### Conformational transition

We computed the feasible conformational transition pathway from open (chain A) to closed (chain H) GroEL monomer structures of the 1sx4 PDB entry. Both conformations are separated by a large conformational gap of 15 Å Cα-RMSD (local superimposition). Approximately 8 min after submitting the corresponding structures, the initial RMSD smoothly decreased to only ∼1 Å (Figure 3). The computed trajectory reasonably illustrates the dramatic conformational changes occurring after ATP, substrate and GroES binding. Such motions involve the concerted twisting and upward rotation of the A domain, which is likely to enable GroES binding and substrate release into the chaperonin cavity. In addition, a feasible conformational transition pathway between the complete *trans (chains from H to N)* and *cis (chains from A to G)* rings was computed. In this case, the functional motion was also captured but RMSD converged to ∼3 Å, which is reasonable considering the restrictions imposed by the other subunits. These GroEL transitions are available in the examples gallery of the server.

### More examples available on the website

In addition to the aforementioned Ca^2+^-ATPase and GroEL examples, the website includes many more illustrative cases. For example, the gallery includes the NMA of the nucleosome to demonstrate the versatility of the approach with a hybrid complex of protein and Deoxyribonucleic acid (DNA). CG strategies are illustrated with two huge viral capsids from the cowpea chlorotic mottle virus and the satellite tobacco mosaic virus. In addition to the external protein capsid, the latter structure contains the viral Ribonucleic acid (RNA). Using similar CG strategies, the bending, stretching and torsion modes of an actin microfilament can be observed. The interesting conformational transition between L and R forms of the bacterial flagellar filament is also provided. Finally, the ability of iMODS to handle homologous structures is demonstrated by making available a feasible pathway between two adenylate kinases structures from different organisms.

## TECHNICAL DETAILS

The web server is implemented as a combination of several PHP, ASP, Perl, Java and JavaScript modules. The computational resources consist of a dedicated Linux system with two Intel Xeon E5-2650 processors running at 2.00 GHz (16 cores) and equipped with 128 GBytes of RAM. For optimal web server usage, a batch queue system is included for job management and scheduling. The web interface is intuitive and responsive to all major browsers and new mobile devices. Modal animations and trajectories can be readily visualized in 3D with JSmol (4) and three different interfaces: Java, HTML5 and WebGL. The computed results can be downloaded, including modes, structures, animations, trajectories, covariance maps, mobility and deformability profiles and affine-model arrows and clusters. Full documentation is provided, including pre-computed examples and tutorials.

## CONCLUSION

The first publicly accessible server for fast NMA in internal coordinates is presented here. Our new server differs from others in the following key aspects: (i) a unique implementation of NMA in dihedral coordinates, which extends efficiency and implicitly maintains stereochemistry; (ii) multi-scale modeling with different atomic representations and elastic networks; (iii) high versatility to handle large heterogeneous systems of proteins and nucleic acids; and (iv) enhanced visualization of collective modes, including an affine model-based representation. Given these improvements, we believe that iMODS will become an appealing and useful tool to explore the collective motions of biological macromolecules.

### Accession Numbers

PDB IDs: 1su4, 1iwo and 1sx4.
